# Platelet‐activating factor: a potential therapeutic target to improve cancer immunotherapy

**DOI:** 10.1002/1878-0261.13758

**Published:** 2024-11-19

**Authors:** Qi Yan, Hemn Mohammadpour

**Affiliations:** ^1^ Department of Cell Stress Biology Roswell Park Comprehensive Cancer Center Buffalo NY USA

**Keywords:** immune therapy, MDSC, neutrophil, PAF, tumor microenvironment

## Abstract

The tumor microenvironment (TME) fosters cancer progression by supporting the differentiation and proliferation of myeloid‐derived suppressor cells (MDSCs), which play a critical role in suppressing immune responses and facilitating tumor growth. Recent findings by Dahal et al. reveal that platelet‐activating factor (PAF), a lipid mediator elevated in the TME, contributes to the differentiation of neutrophils into immunosuppressive neutrophils. They showed that inhibiting PAF signaling reduces MDSC‐mediated immunosuppression, thereby enhancing cytotoxic T‐cell activity. This approach may improve cancer immunotherapy outcomes, particularly when combined with checkpoint blockade therapies, suggesting a promising avenue for therapeutic development.

## Graphical abstract

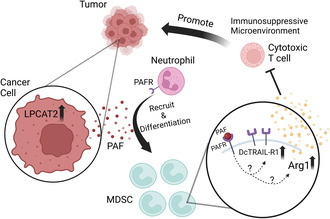

**Abbreviations**
Arg1: arginase‐1
MDSCs: myeloid‐derived suppressor cells
M‐MDSCs: monocytic myeloid‐derived suppressor cells
PAF: platelet‐activating factor
PAFR: platelet‐activating factor receptor
PMN‐MDSCs: polymorphonuclear myeloid‐derived suppressor cells
TME: tumor microenvironment

## 1 Tumor microenvironment‐mediated MDSC differentiation

The tumor microenvironment (TME) forms a complex and dynamic setting that plays a crucial role in cancer progression and immune escape. A critical component of this environment is myeloid‐derived suppressor cells (MDSCs), which establish an immunosuppressive landscape that supports tumor growth and metastasis [1]. MDSCs, a diverse cell population, are classified into two primary subtypes: polymorphonuclear (PMN‐MDSCs), which resemble neutrophils, and monocytic (M‐MDSCs), similar to monocytes [2]. The differentiation and accumulation of MDSCs within the TME is a result of interactions from a range of tumor‐derived factors—including cytokines, growth factors, and lipid mediators—that collectively create an environment conducive to the recruitment and maturation of immature myeloid cells into MDSCs [3].

Key cytokines and growth factors secreted by tumor and stromal cells within the TME drive this process of MDSC differentiation and expansion. For instance, granulocyte‐macrophage colony‐stimulating factor (GM‐CSF), granulocyte colony‐stimulating factor (G‐CSF), interleukin‐6 (IL‐6), interleukin‐1β (IL‐1β), and vascular endothelial growth factor (VEGF), are all recognized for their roles in promoting MDSC expansion [4]. GM‐CSF and G‐CSF are particularly vital for transforming myeloid progenitor cells into PMN‐MDSCs [5]. IL‐6, a widely acting pro‐inflammatory cytokine, enhances MDSC differentiation through activation of the STAT3‐ERK1/2 signaling pathway, which supports MDSC survival and immunosuppressive function [6, 7]. IL‐1β also promotes MDSC differentiation by triggering the secretion of chemokines like CXCL1 and CXCL2, which attract MDSCs to the tumor site, thus reinforcing the tumor‐promoting functions within the TME [8].

## 2 Platelet‐activating factor: function, therapeutic potential, and challenges

**Fig. 1:**
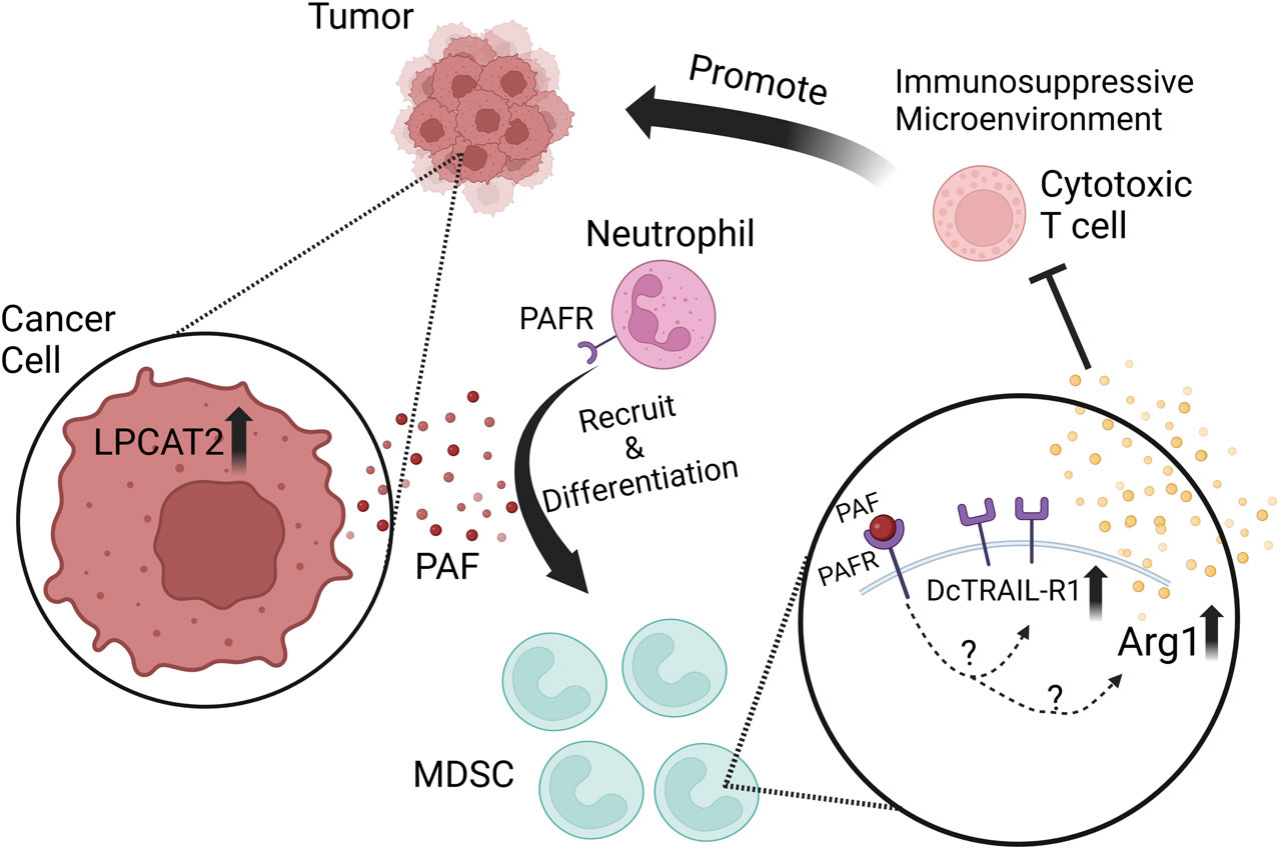
Cancer cells with upregulated LPCAT2, an enzyme for PAF synthesis, present increased secretion of PAF. PAF binding to PAFR attracts neutrophils to the tumor site and promotes neutrophil differentiation into immunosuppressive neutrophils. The upregulation of Arginase‐1 (Arg1) and DcTRAIL‐R1 enhances the immunosuppressive function of immunosuppressive neutrophils and promotes their extended survival, resulting in cytotoxic T‐cell suppression and promoting tumor progression.

Among influential mediators in the TME, platelet‐activating factor (PAF) stands out as a potent lipid mediator with a dual role in inflammation and immune modulation. PAF is involved in many physiological processes, including wound healing, inflammation, reproduction, and angiogenesis [9]. Emerging research by Dahal et al. elucidates PAF's role in driving neutrophils to differentiate into immunosuppressive neutrophils within solid tumors. The study reveals that PAF secretion by tumor cells not only attracts neutrophils to the tumor site but also directs their differentiation into immunosuppressive neutrophils through PAF‐PAF receptor (PAFR) signaling. This pathway supports the upregulation of immunosuppressive factors like Arginase‐1 (Arg1) and DcTRAIL‐R1. DcTRAIL‐R1 is a decoy TRAIL receptor which can block TRAIL‐induced apoptosis [10]. It is reported that upregulation of DcTRAIL‐R1 can prolong the lifespan of tumor‐associated neutrophils [11]. Thus, the upregulation of the factors can enhance the survival and immune suppressive function of immunosuppressive neutrophils, thereby aiding tumor progression by inhibiting cytotoxic T‐cell activity (Fig. 1).

By employing advanced lipidomics, Dahal et al. [12] further confirm that various tumor types—spanning murine and human models—exhibit high PAF levels, positioning PAF as a widespread mediator of immune suppression within the TME. This finding has significant therapeutic implications, as targeting PAF pathways may offer a novel approach to counteract the immunosuppressive TME. In fact, the study demonstrates that using a PAFR antagonist (WEB2086) reduces PMN‐MDSC activity and, correspondingly, tumor growth in preclinical models. This promising approach could potentially reinvigorate cytotoxic T‐cell responses, thereby enhancing the efficacy of current cancer immunotherapies like checkpoint inhibitors.

In the context of human cancers, PAF's clinical relevance extends further. Dahal et al. [12] report a correlation between the expression of LPCAT2 (an enzyme crucial for PAF synthesis) and increased neutrophil infiltration in patient tumors. This suggests PAF could serve as a biomarker for aggressive cancer phenotypes and poor clinical outcomes, making it a valuable target for therapeutic exploration. Despite these promising insights, challenges remain. PAF is involved in several physiological processes, including platelet aggregation and inflammation [13]. Therefore, systemic targeting of PAF may carry potential risks of unintended effects such as delayed wound healing or an increased vulnerability to infections [14]. This necessitates a nuanced understanding of PAF‐mediated pathways in MDSC differentiation to design targeted, safe, and effective interventions.

Furthermore, PAF's broader role in immune modulation is not yet fully understood. While Dahal et al. [12] focus on PAF's influence on neutrophil‐to‐PMN‐MDSC differentiation, the potential effects on other immune cells, such as M‐MDSCs and regulatory T‐cells (Tregs), remain to be investigated [15]. Understanding these broader implications could refine PAF‐targeted therapies and enhance their effectiveness. Additionally, combining PAF antagonists with other immunotherapies, such as checkpoint inhibitors, holds significant promise. Since MDSCs often limit the efficacy of checkpoint inhibitors, a combination approach may reduce immunosuppression within the TME, leading to more robust and sustained patient responses.

## 3 Conclusion

In conclusion, the research by Dahal et al. underscores PAF's central role in fostering an immunosuppressive TME by driving the differentiation of neutrophils into immunosuppressive neutrophils. By identifying PAF as a potential therapeutic target, this study opens a promising avenue for cancer treatment, suggesting that PAF inhibition, possibly in combination with other immunotherapies, could effectively dismantle the tumor‐favoring immune environment. Future research should prioritize optimizing PAF‐targeted therapies, exploring synergistic treatment combinations, and carefully evaluating the broader physiological impacts of PAF inhibition. With continued progress, targeting PAF could represent a powerful new strategy in the fight against cancer.

## Conflict of interest

The authors declare no conflict of interest.

## Author contributions

QY and HM wrote and edited the commentary.
